# A 5G NR FR2 Beamforming System with Integrated Transceiver Module

**DOI:** 10.3390/s24061983

**Published:** 2024-03-20

**Authors:** Ayush Bhatta, Md Kamrojjaman, Sanghoon Sim, Jeong-Geun Kim

**Affiliations:** 1 Department of Electronic Engineering, Kwangwoon University, Seoul 01897, Republic of Korea; ayush@kw.ac.kr (A.B.); kamrojjamandiu@kw.ac.kr (M.K.); 2 School of Electronics Engineering, Chungbuk National University, Cheongju 28644, Republic of Korea; shsim@cbnu.ac.kr

**Keywords:** 5G, beamforming system, transceivers, phased array, RFIC, mm-wave

## Abstract

This paper presents a 5G new radio (NR) FR2 beamforming system with an integrated transceiver module. A real-time operating module providing enhanced flexibility and capability has been proposed. The integrated RF beamforming system with an integrated transceiver module can be operated in 8Tx-8Rx mode configuration simultaneously. A series-fed structure 8 × 7 microstrip antenna array for compact size and improved directivity is employed in the RF beamforming module. The RF beamforming module incorporates a custom 28 GHz, eight-channel fully differential beamforming IC (BFIC). An eight-channel BFIC in a phased-array beamforming system offers advantages in terms of increased antenna density and improved beam steering precision. The RF beamforming module is integrated with an RF transceiver module that enables the simultaneous up-conversion and down-conversion of the baseband signal. The RF transmitter module consists of a transmitter, a receiver, a signal generator, a power supply, and a control unit. The RF beamforming system can scan horizontally from −50° to +50° with a step of 10°. To achieve an optimized beam pattern, a calibration was conducted. The transmit and receive conversion gain of around 20 dB is achieved with the transceiver module. To verify the communication performance of the manufactured integrated RF beamforming system, a real-time wireless video transmission/reception test was performed at a frequency of 28 GHz, and the video file was transmitted smoothly in real time without interruption within a range of ±50°.

## 1. Introduction

In recent years, the rapid evolution of wireless communication technologies has paved the way for transformative advancements in connectivity solutions. At the forefront of this revolution are the developments in the fifth-generation (5G) wireless communication systems, which have set new benchmarks for speed, latency, and reliability [1,2,3,4,5,6,7]. However, as the demand for higher data rates and seamless connectivity continues to escalate, there arises a pressing need for even more sophisticated and efficient systems. This imperative has led to the emergence of beyond 5G (B5G) technologies, representing the next frontier in wireless communication evolution [8,9,10,11]. In the era of B5G, the advancements in wireless communication technology are expected to support the emergence of new industries that heavily rely on mobile vehicles such as autonomous vehicles, robots, and drones. Additionally, the widespread adoption of virtual reality (VR), augmented reality (AR), and mixed-reality (MR) technologies, which leverage high-resolution imaging, is anticipated. These immersive technologies rely on high-resolution imaging and real-time data streaming, requiring a communication infrastructure capable of delivering high-quality content with minimal latency [12,13,14,15,16]. Hence, mm-wave communication systems have played a pivotal role in achieving high data rates, benefiting from the extensive available spectrum at higher frequencies. The key enablers for these systems are phased arrays based on multi-channel CMOS beamformer ICs, offering higher levels of integration and lower costs [17,18,19,20,21]. An antenna array with a large number of elements is needed to electronically steer the directional beams toward the desired directions in mm-wave systems. This is achieved by controlling the phase and magnitude of signals at each antenna based on beamforming techniques.

In this paper, the development of a 28 GHz 8 × 7 RF beamforming phased-array system with an integrated transceiver module is proposed. The development of a 5G beamforming system carries its own novelty and significance, particularly considering the ongoing deployment and commercialization of 5G wireless communication. As mm-wave communication systems are still on the verge of being fully implemented, the development of a complete beamforming system specifically designed for 5G is not yet a common occurrence. In this work, we demonstrate a circuit to the system. The integration of our own eight-channel BFIC, array antenna, and our own measurement setup enables the effective demonstration of a 5G mm-wave system. This system aims to address the challenges posed by the B5G era, including the need for ultra-low latency, high-speed movement, and reliable communication in dynamic environments. The 28 GHz RF beamforming system can dynamically steer the antenna array’s radiation pattern through electronic means, allowing for real-time adjustments based on environmental conditions. This functionality enhances both performance and efficiency, ensuring optimal operation [22,23,24,25]. Moreover, the integrated transceiver module possesses the flexibility to easily adjust the frequency of both the local oscillator (LO) and intermediate frequency (IF) with minimal complexity. This capability allows the implementation of communication systems and radars in the millimeter-wave range band, enabling the swift testing of innovative solutions utilizing software-defined radio. The developed RF beamforming system incorporates a series-fed structure 8 × 7 microstrip antenna array and a custom 28 GHz, eight-channel fully differential BFIC. The 28 GHz RF transceiver module was manufactured using commercially available off-the-shelf (COTS) chipsets. The integrated module can operate with 8Tx-8Rx mode simultaneously. In particular, the 28 GHz RF beamforming system and transceiver module offer simultaneous data transmission and reception using the same module. This proposed beamforming system may hold promise for diverse future applications, including 5G communications, radar uses, IoT, and surveillance scenarios.

The remainder of this paper is organized as follows: Section 2 presents the design and development of the 28 GHz RF beamforming system and transceiver module. Section 3 provides the fabrication and measurement of the beamforming system and transceiver module and verifies the beamforming system operation by transmitting real-time videos in a different direction. Finally, Section 4 concludes this article.

## 2. A 28 GHz RF Beamforming System and Transceiver Module

**Figure 1:**
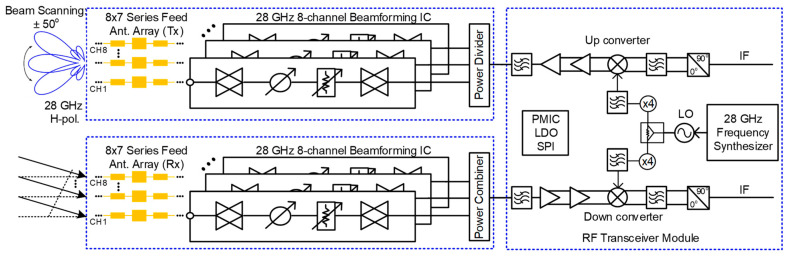
Block diagram of the proposed 28 GHz RF beamforming system and transceiver module.

Figure 1 shows the block diagram of the proposed 28 GHz RF beamforming system and transceiver module. The block diagram is divided into two parts, the RF beamforming antenna system and the RF transceiver module.

### 2.1. Development of 28 GHz RF Beamforming System

**Figure 2:**
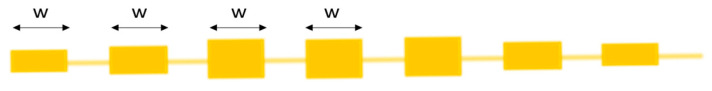
Layout of 1 × 7 series-fed microstrip patch antenna.

**Figure 3:**
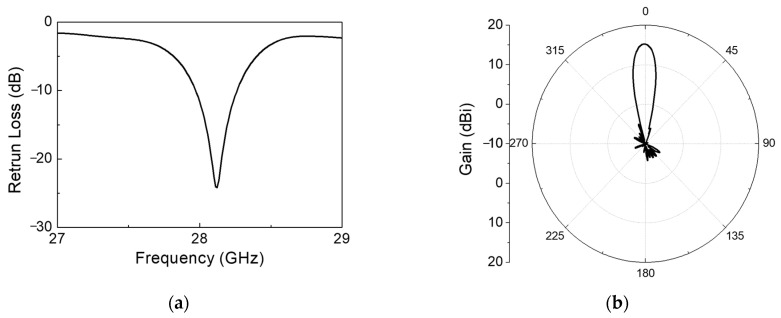
Simulated 1 × 7 series-fed antenna results: (**a**) return loss, (**b**) E-plane radiation pattern.

According to antenna array theory, a linear array can achieve a minimized sidelobe level (SLL) by tuning the amplitude and phase of individual elements. The 28 GHz RF beamforming system employs a 1 × 7 series-fed microstrip patch antenna capable of designing a phased-array antenna with low beam width using CMOS BFIC, although beamforming is only possible in the horizontal direction. Figure 2 shows the geometry of the 1 × 7 series-fed microstrip patch antenna linear array. In the case of a series-fed patch antenna array, the amplitude is associated with the patch width, while the phase is linked to the element spacing. Consequently, achieving a low SLL involves the tapering of the patch width and introducing unequal spacing between elements. The series-fed microstrip patch antenna boasts advantages such as its compact size and enhanced directivity, making it well-suited for beamforming applications. The size of the series-fed microstrip antenna is determined using the Chebyshev weighting method and manufactured using a low-loss RF PCB substrate. The seven patch elements with the same length w are arranged symmetrically, and they are connected by a narrow microstrip line. Figure 3a shows simulated return loss results of the 1 × 7 series-fed antenna. The return loss of the antenna obtained is over −10 dB at 28 GHz. Figure 3b shows the 2D E-plane radiation pattern of the antenna at 28 GHz center frequency. The antenna gain is 15 dBi and the 3 dB beam width is 15°.

**Figure 4:**
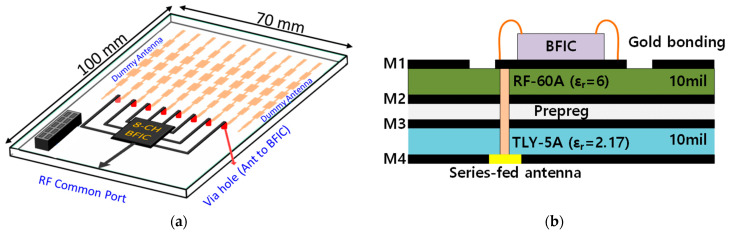
(**a**) Proposed RF beamforming antenna system. (**b**) Cross-section of RF PCB with the bonding.

**Figure 5:**
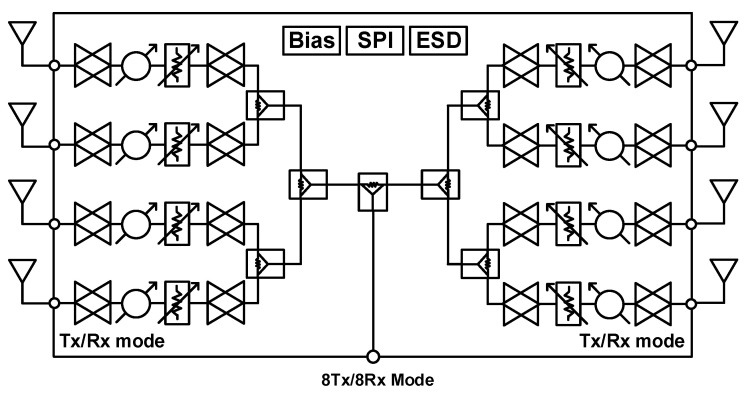
Block diagram of 28 GHz eight-channel multimode BFIC.

**Figure 6:**
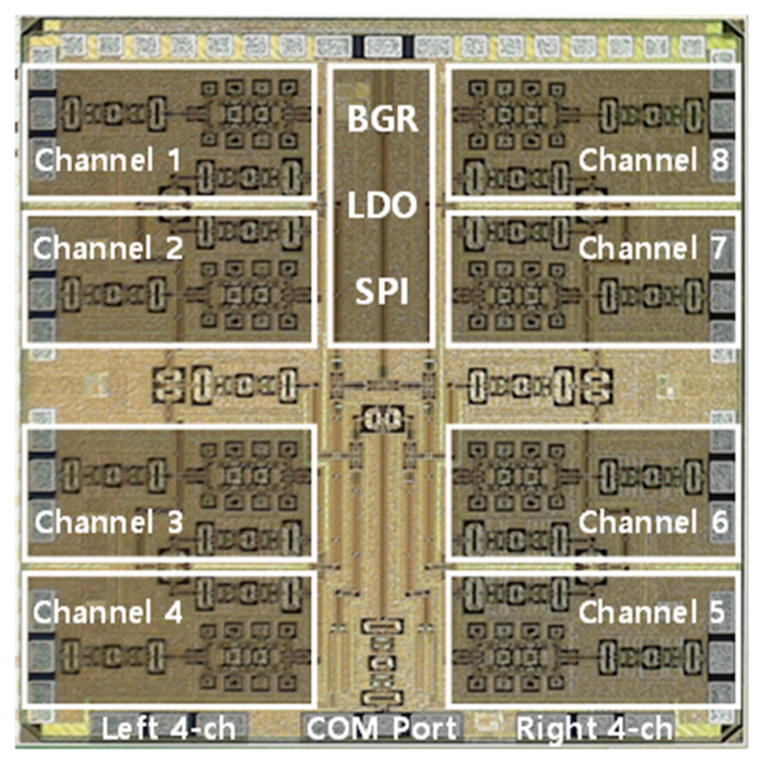
Microphotograph of the 28 GHz eight-channel multimode BFIC [26].

The proposed block of the 28 GHz RF beamforming system is shown in Figure 4a. The beamforming system uses an RF low-loss PCB, and the cross-section of RF PCB with chip bonding is shown in Figure 4b. An 8 × 7 phased-array antenna is manufactured using a 1 × 7 series-fed microstrip patch antenna. The spacing of each antenna is 5 mm at 28 GHz. The planar-type phased-array antenna connects the RF and series-fed patch array antenna using a hole for signal feeding. Equations (1) and (2) show that the antenna gain and the EIRP are directly proportional to the number of elements in the array. The 28 GHz RF beamforming system incorporates an eight-channel fully differential beamforming IC. This implies that eight antennas can be connected simultaneously for either 8Tx (transmit) or 8Rx (receive) mode separately, as all eight channels can operate bidirectionally, resulting in a narrow beam, high EIRP, and improved SNR. This configuration allows for the versatile operation and efficient utilization of antenna resources for transmission and reception. The simplified block diagram of the eight-channel TRX beamforming IC is shown in Figure 5. The 28 GHz eight-channel CMOS beamforming chip was mounted on a PCB using Chip-on-Board (COB) technology. The microphotograph of the 28 GHz eight-channel fully differential CMOS BFIC is shown in Figure 6. Each channel of an eight-channel fully differential CMOS T/R IC consists of six-bit differential phase shifters, three-bit differential attenuators, differential two-stage bi-directional amplifiers (BDAs) with variable gain control, a differential two-way power divider/combiner, and an SPI. The tuning bits of 5.6°, 1 dB are placed in the PS/ATT blocks to compensate for the phase and amplitude errors. A shift registers to control the phase shifter and attenuator including tuning bits, and the two-stage bi-directional amplifiers make phase and gain controls easy. In addition, the bias circuits including BGR and LDO are integrated to provide a stable DC bias.

(1) $$Antenna\;Gain=\frac{Radiation\;intensity\;in\;desired\;direction}{\begin{array}{c} Radiation\;intensity\;of\\ \;isotropic\;antenna\;(all\;angles) \end{array}}=10LogN+Ge$$

(2) EIRP=Pt×Gt

where N is the number of elements, Ge is the element gain, Gt is the antenna gain, and Pt is the total transmitted power.

**Figure 7:**
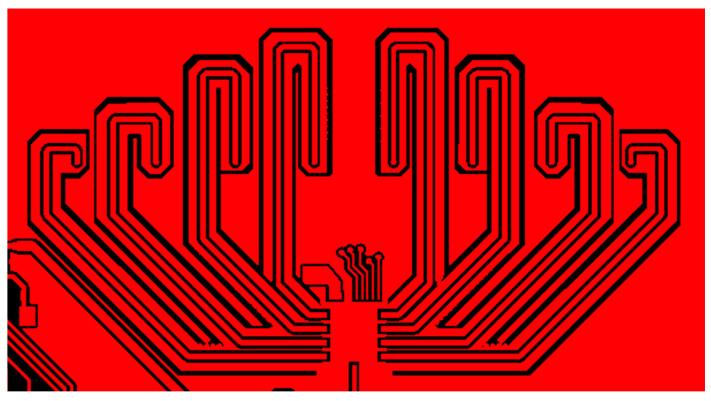
Feedline layout of RF line connecting the BFIC and the series-fed antenna.

**Figure 8:**
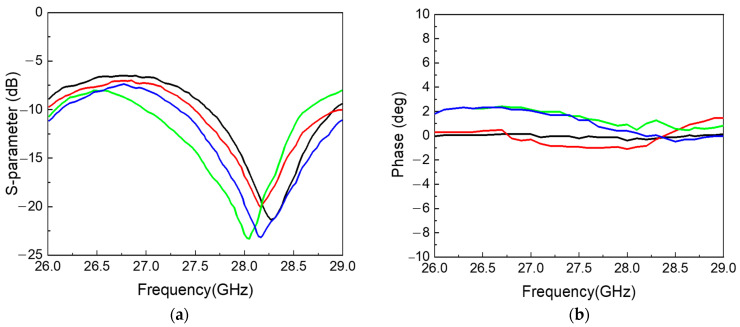
EM simulation results of feedline: (**a**) return loss, (**b**) phase characteristics.

The eight channels of the fully differential BFIC are connected to the respective antenna through the feeding line. All the RF feeding lines are designed with grounded CPW lines to prevent unwanted coupling by adjacent channels. The layout of the feeding lines connecting each channel of BFIC and the respective series fed antenna is shown in Figure 7. The whole feedline length is equal, and the feedline CPW ground is isolated from the overall ground to improve the performance. The EM simulation of all the feedlines is performed to ensure the loss and the same phase of the feedline. The return losses of the feedline are over −10 dB at a frequency of 27.5 GHz to 29 GHz, as shown in Figure 8a. The phase difference in all the feedlines is around 1° at 28 GHz, as shown in Figure 8b. The eight-channel BFIC integrates an SPI. This SPI facilitates both phase and amplitude controls directly on the chip. The SPI controls are wire bonded from the chip to connect the connector in the PCB. Hence, the beamforming module includes SPI interfaces for BFIC control and DC power connectors. A non-soldered K-type connector with a small form factor was used to measure the antenna beam pattern.

### 2.2. Development of 28 GHz RF Transceiver Module

The 28 GHz RF transceiver module consists of an up-converter, down-converter, phased locked loop (PLL) frequency synthesizer, and power and digital controller. In the transceiver module, we used the commercially available chips for the up-converter (ADMV 1013), down-converter (ADMV 1014), and PLL (ADF 4355) from the analog device (Analog Devices, Wilmington, MA, USA). A mixer-based RF transceiver structure was employed to enable simultaneous communication and sensing in the millimeter-wave range by up-converting and down-converting the baseband signal to the millimeter-wave frequency. The 28 GHz RF transceiver used a super-heterodyne method. The RF signal generator was implemented in the form of a frequency synthesizer, based on a PLL, capable of various frequency generations with low spurious content and excellent phase noise characteristics. The upconverter integrated a mixer, RF power amplifier, RF voltage variable attenuator (VVA), and LO frequency quadrupler. An external 90° hybrid coupler was used to achieve single-band up-conversion and to suppress the signal in unwanted sidebands. Similarly, the down-converter integrated the LNA, mixer, IF PA, and LO frequency quadrupler, resulting in the signal being received independently. The up-converter and down-converter provide two modes of frequency translation. The up-converter can directly convert in-phase quadrature (I/Q) input signals from baseband to RF. Additionally, it supports single-sideband (SSB) up-conversion when given complex intermediate frequency (IF) inputs. Similarly, the down-converter is capable of direct quadrature demodulation to the baseband in-phase (I)/quadrature (Q) output signals, as well as image rejection down-conversion to a complex intermediate frequency (IF) output carrier frequency. The assumption of the input signal with image signal and LO source can be expressed as follows:

(3) $$v_{in}\left(t\right)=A_{RF}cos\left(w_{RF}t+\Phi_{RF}\right)+A_{Img}cos\left(w_{Img}t+\Phi_{Img}\right)$$

(4) $$v_{LO\_I}\left(t\right)=sin(w_{LO}t+\Phi_{LO})$$

(5) $$\;v_{LO\_Q}\left(t\right)=cos(w_{LO}t+\Phi_{LO})$$

Then, the mixed signal can be expressed as

(6) $$v_{I}\left(t\right)={-\frac{1}{2}A}_{RF}sin\left(w_{IF}t+\Phi_{RF}-\Phi_{LO}\right)+\frac{1}{2}A_{Img}sin\left(w_{IF}t+\Phi_{LO}-\Phi_{IMG}\right)$$

(7) $$v_{Q}\left(t\right)={-\frac{1}{2}A}_{RF}cos\left(w_{IF}t+\Phi_{RF}-\Phi_{LO}\right)+\frac{1}{2}A_{Img}cos\left(w_{IF}t+\Phi_{LO}-\Phi_{IMG}\right)$$

The image signal is mitigated by employing an external 90° hybrid coupler, and the resulting intermediate frequency (IF) output can be expressed as follows:

(8) $$v_{IF}\left(t\right)=A_{RF}cos\left(w_{IF}t+\Phi_{RF}-\Phi_{LO}\right)$$

The IF frequency ranges from 0.8 GHz to 4.5 GHz. The frequency range for creating the LO signal ranges from 5.6 GHz to 6.4 GHz. Therefore, the RF band spans from 24.25 GHz to 30.1 GHz. To generate a frequency in the 28 GHz band, a frequency multiplier was built into the transmitter and receiver chip, so the PLL could operate at a frequency lower than 28 GHz, reducing the power loss when distributing for transmitting and receiving LO supply. In order to supply the LO signal to the transceiver, differential outputs were supplied to the transmitter and receiver one by one. The transceiver module was mainly based on the commercially available RF surface-mounted components from the analog devices including the up-converter module, down-converter module, and local oscillator (LO) source module.

The IF signal supplied was connected using an SMA connector, and to link the 28 GHz band beamforming antenna, the RF transceiver integrated board was equipped with an RF/DC connector for power supply and SPI control, and a K-type connector was used. This reduced the insertion loss between the beamforming antenna and the RF transceiver. In order to supply stable power to the transceiver module and reduce electrical signal interference between the block, the power supply and ground were separated by the functional block. Additionally, to simplify the digital control of the RF chipsets, the use of SPI was employed.

## 3. Fabrication and Measurement

### 3.1. Measurement of 28 GHz RF Beamforming System

**Figure 9:**
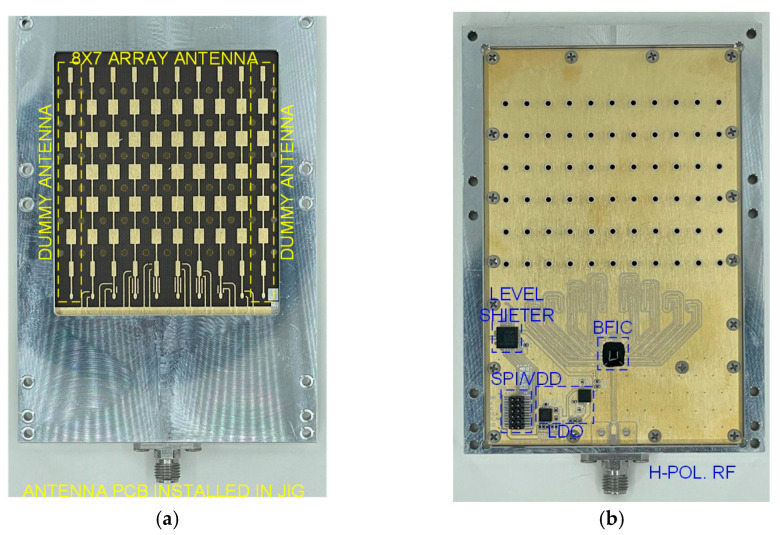
(**a**) Antenna-side view and (**b**) chip-side view of 28 GHz phased-array beamforming antenna module using eight-channel CMOS BFIC.

To develop a highly directional beamforming antenna, an array antenna with a series-fed structure was fabricated using a low-loss RF PCB substrate. Figure 9a shows the antenna-side view of the assembled 28 GHz 8 × 7 phased-array antenna beamforming module using an eight-channel CMOS BFIC, and Figure 9b shows the chip-side view of the beamforming antenna module. All the RF lines connecting the antenna and the BFIC were equal in length. An array of dummy antenna surrounded the 8 × 7 array for improved antenna impedance at large scan angles. The spacing between each antenna was 5 mm at 28 GHz. To measure the antenna beam pattern, a non-soldered K-type connector with a small form factor was used. A test pattern of the RF beamforming system was created to integrate the SPI interface and DC power connector for beamforming chip control. The fabricated beamforming antenna measured 100 × 70 × 70 mm^3^. The beamforming antennas were enclosed in the aluminum structure to minimize heat dissipation and establish a secure ground connection.

**Figure 10:**
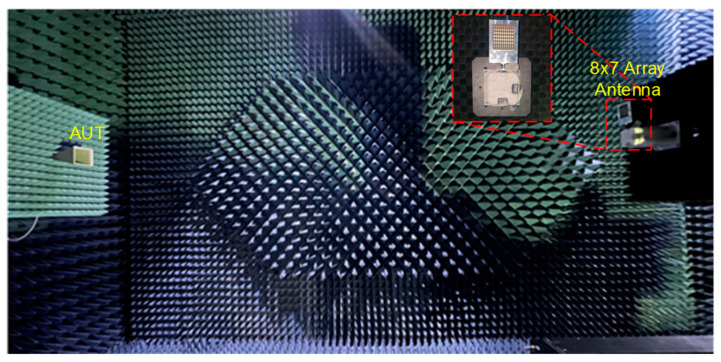
Measurement setup for 28 GHz 8 × 7 beamforming antenna module in anechoic chamber.

**Figure 11:**
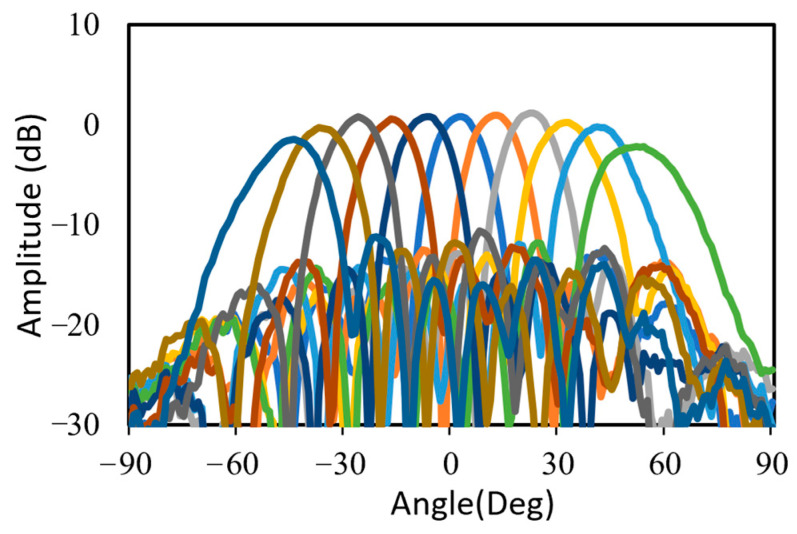
Normalized measured TX radiation patterns scanning up to ±50° at 28 GHz.

The far field calibration method was used to calibrate each channel of the beamforming antenna. The calibration process involved individually activating each channel to verify the phase and attenuation of the antennas within the array. Following this, adjustments were made to the phase of each antenna element using the tuning phase bits of the beamforming IC. The 28 GHz RF beamforming system was measured in a far-field anechoic antenna chamber with a standard horn antenna, as shown in Figure 10. The phase shifter and attenuator of the eight channels was controlled by the SPI with far-field measurement configuration. The antenna beam was scanned at a step of 10° intervals within a ±50° Field of View (FOV) at the frequency of 28 GHz. The variable attenuator of the beamforming chip was adjusted to apply Chebyshev weighting, resulting in sidelobe suppression of 15 dB. A half-power beam width (HPBW) of 13° was achieved. The normalized measured TX radiation patterns scanning up to ±50° at 28 GHz are shown in Figure 11. Table 1 summarizes the performance of the 28 GHz RF beamforming system and compares it with previously published beamforming modules.

### 3.2. Measurement of 28 GHz RF Transceiver Module

**Figure 12:**
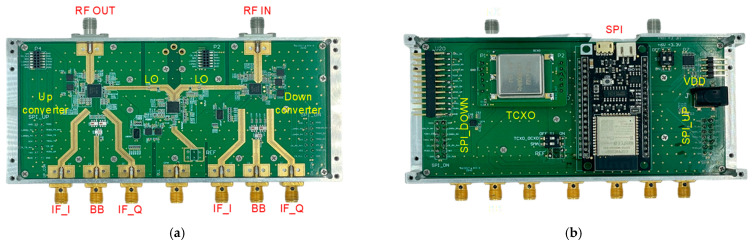
Manufactured 28 GHz RF transceiver module: (**a**) top view and (**b**) bottom view.

The integrated 28 GHz RF transceiver module was manufactured using commercially available off-the-shelf (COTS) chipsets and RF passive components such as RF switches, frequency up/down-converters, and millimeter-wave signal sources. To fabricate the 28 GHz RF transceiver module, the performance of the individual RF circuit chips was validated, and the design and performance of RF passive components on a PCB were verified. Especially at the high frequencies of the 28 GHz band where RF performance degradation often occurs during SMT, actual measurements were conducted to compare and analyze the performance against the datasheet specifications. Figure 12a shows the top view of the manufactured 28 GHz RF transceiver module, and Figure 12b shows the bottom view of the transceiver module. To minimize power consumption and losses, a frequency conversion structure utilizing a frequency quadrupler at the LO stage was employed in the frequency up/down-converters. To facilitate LO signal supply and frequency variability, a frequency synthesizer in the form of a PLL chip was utilized. To supply the LO signal to the transceiver, differential outputs were supplied to the transmitter and receiver one by one. The RF transceiver board was manufactured using a cost-effective multilayer FR4 PCB. The interface between the RF components utilized the Grounded Coplanar Waveguide (CPWG) transmission lines to minimize losses and coupling between adjacent lines. To implement the RF transceiver on a single PCB, the isolation between the transmission and reception was crucial. The RF signals, baseband signals, and control signals were separated into different groups in the PCB. The size of the fabricated RF transceiver module was 103 × 60 × 38 mm^3^, implemented with an input voltage of 6V and a current consumption of 1.4 A. A command-based control program was developed using SPI and UART for gain/power/frequency variability control.

**Figure 13:**
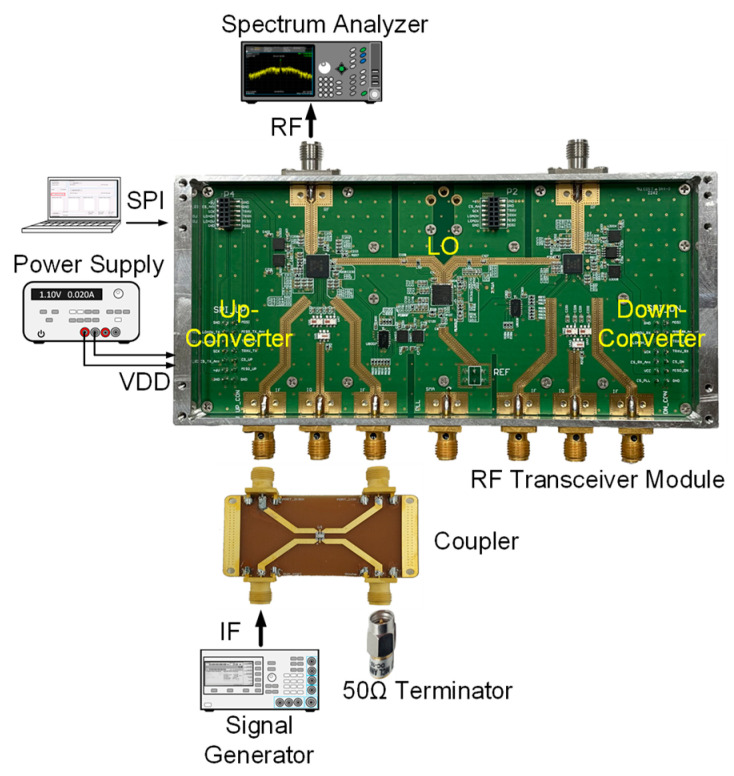
Measurement setup of 28 GHz frequency up-converter.

**Figure 14:**
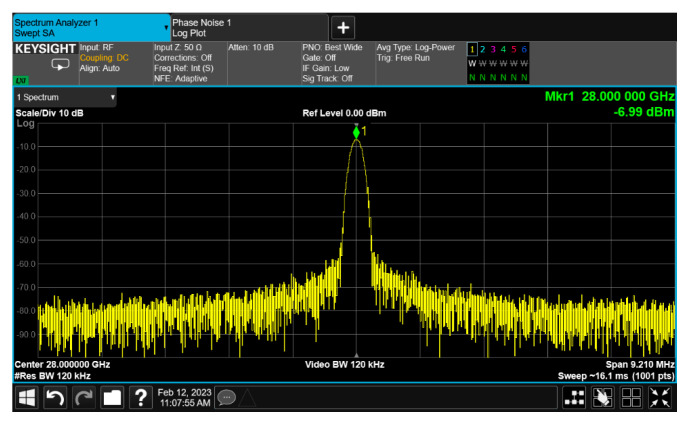
RF transceiver up-converter output signal measurement at 28 GHz.

**Figure 15:**
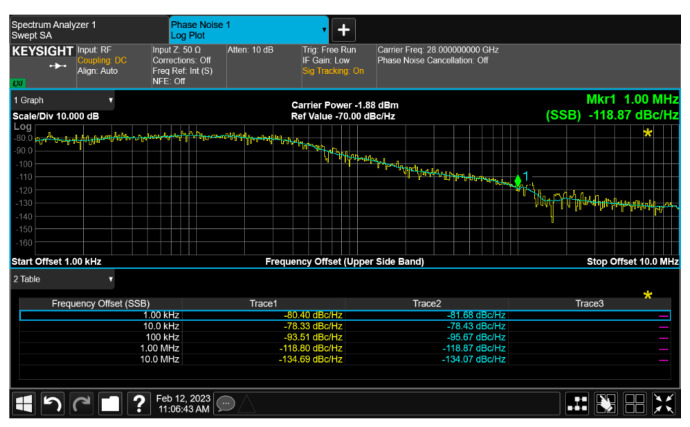
RF transceiver up-converter phase noise at 28 GHz.

**Figure 16:**
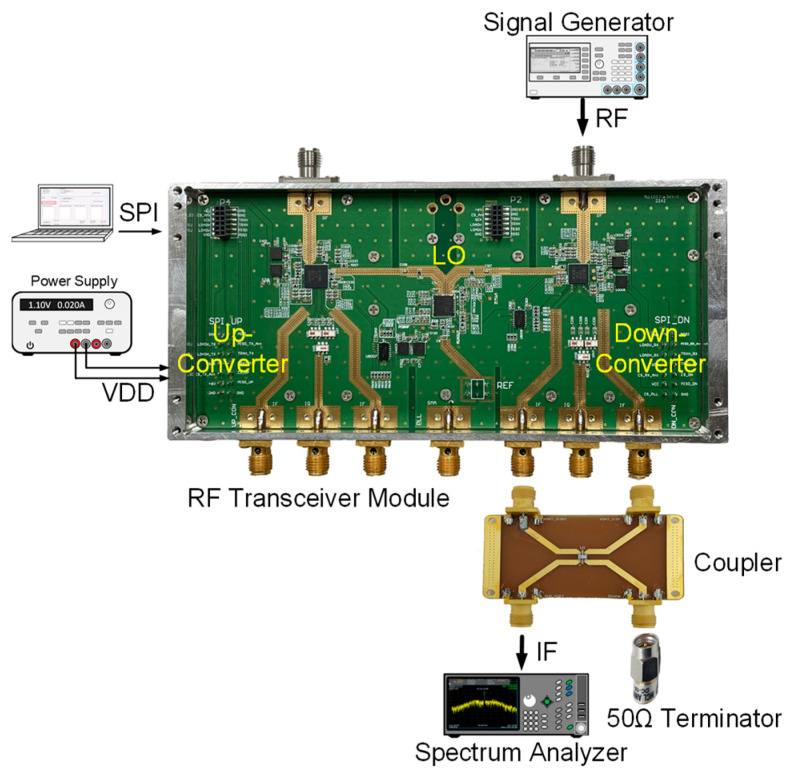
Measurement setup of 28 GHz frequency down-converter.

**Figure 17:**
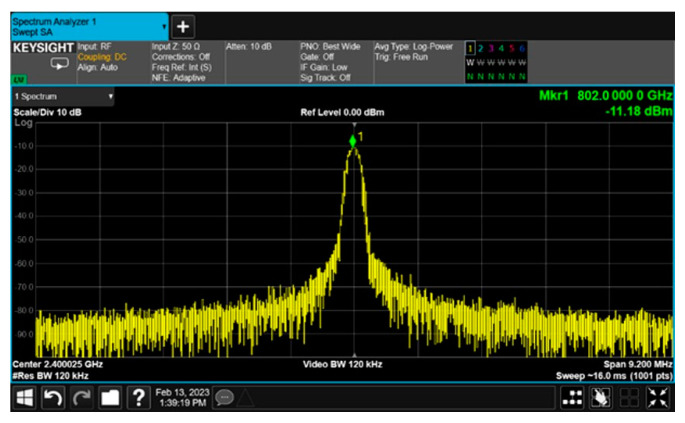
RF transceiver down-converter output signal measurement at 802 MHz.

The up-converter measurement of the RF transceiver was performed by supplying the IF frequency of 802 MHz through the signal generator. An external 90° hybrid coupler was used to select the appropriate sideband for conversion and suppress the signal in the unwanted sideband. The measurement setup of the 28 GHz up-converter is shown in Figure 13. The LO of the transmitter applied a 0 dBm signal at a frequency of 6.8 GHz, and a quadrupler was built inside the transmitter, so the LO frequency of the actual frequency converted was applied at 27.2 GHz. At this time, it was confirmed that the RF signal was output at a frequency of 28 GHz, as shown in Figure 14. The image and the LO signals were highly attenuated compared to the RF signal. At this time, the phase noise at a 1 MHz offset frequency was measured to be −118.87 dBc/Hz at 28 GHz, as shown in Figure 15. The frequency conversion gain of the transmitter was measured to be around 20 dB. The down-converter module was designed similarly to the up-converter. The down-converter measurement of the 28 GHz RF transceiver module was performed by supplying the RF frequency of 28 GHz through the signal generator. The measurement setup of the 28 GHz down-converter is shown in Figure 16. An external 90° hybrid coupler was used to select the appropriate sideband. The LO of the receiver applied a 0 dBm signal at a frequency of 6.8 GHz, and a quadrupler was built inside the receiver, so the LO frequency of the actual frequency converted was applied at 27.2 GHz. At this time, the IF signal output at a frequency of 802 MHz was as shown in Figure 17.

### 3.3. Measurement of 28 GHz Integrated RF Beamforming System and Transceiver Module

#### Real-Time Video Transmission Experiment

**Figure 18:**
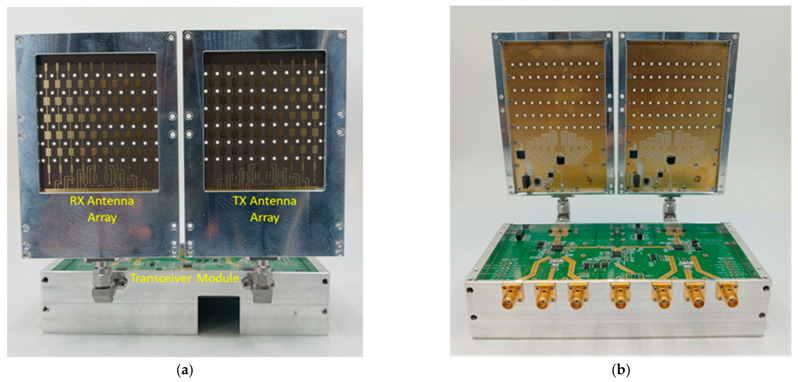
Integrated 28 GHz RF beamforming system and RF transceiver module: (**a**) front view and (**b**) back view.

**Figure 19:**
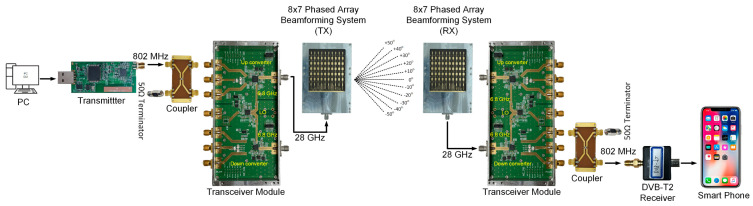
Real-time video transmission experimental setup at 28 GHz.

The manufactured 28 GHz RF beamforming system and transceiver module were combined using a K-type connector. The integrated 28 GHz RF beamforming system and RF transceiver module antenna-side view is shown in Figure 18a, and the back-side view is shown in Figure 18b. The integrated module can operate with 8Tx-8Rx mode simultaneously. In particular, the 28 GHz RF beamforming system and transceiver module offer simultaneous data transmission and reception. To verify the communication performance of the manufactured transmission and reception system, a wireless video transmission and reception experiment was performed at a millimeter-wave frequency of 28 GHz. Figure 19 shows the 28 GHz real-time video transmission and reception test setup of the developed video transmission system. The IF transmitter can transmit and receive videos in TS (Transport Streaming) format through a PC, with the capability to transmit signals up to 900 MHz. The receiver can scan a signal up to 900 MHz and display the video. The high-definition video files at 28 GHz are transmitted smoothly in real time without interruption.

**Figure 20:**
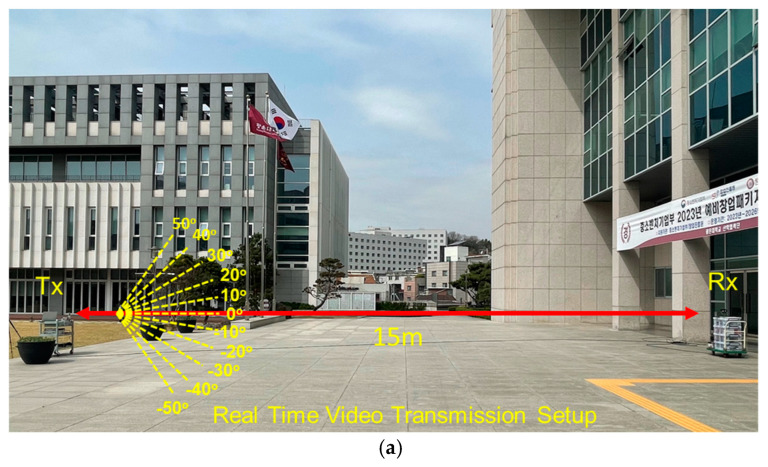

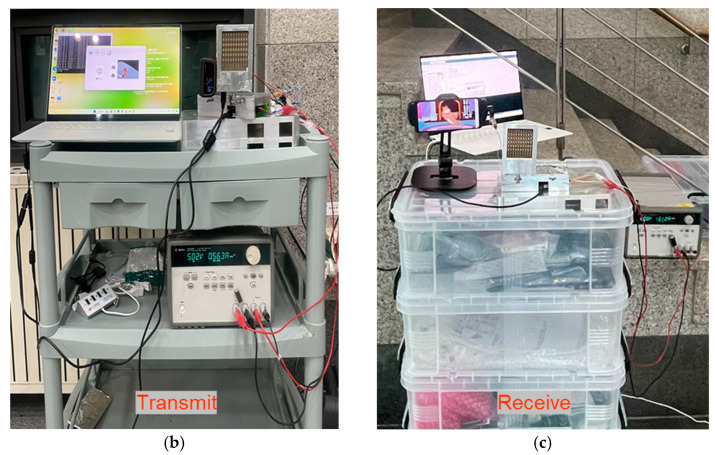
(**a**) The 28 GHz real-time video transmission outdoor experimental setup: (**b**) transmitter and (**c**) receiver.

**Figure 21:**
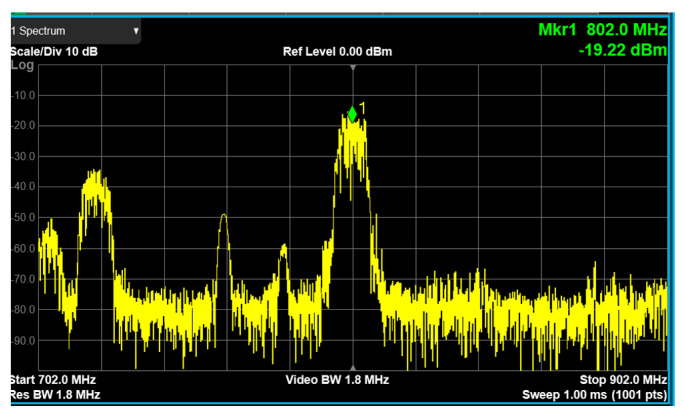
A transmitted OFDM measurement spectrum from 28 GHz RF transceiver.

**Figure 22:**
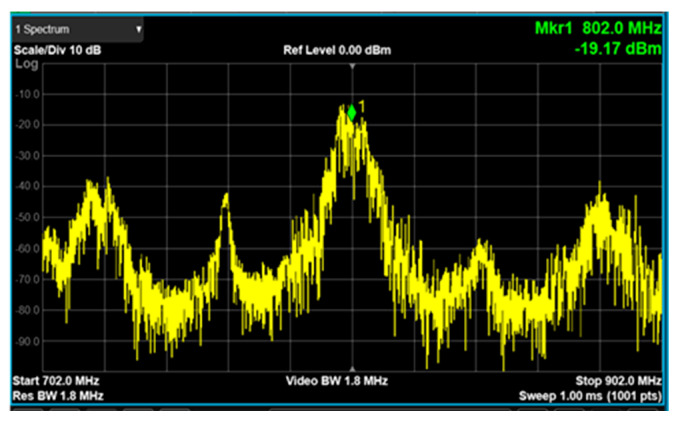
A received OFDM measurement spectrum from 28 GHz RF transceiver.

In the wireless video transmission experiment at 28 GHz, the IF transmitter used a frequency of 802 MHz, which was up-converted to high-frequency signals using the integrated transceiver module proposed in this paper. An IF signal with a frequency of 802 MHz was sourced from a DVB-T system, employing 64 QAM modulation. The LO frequency was set to 6.8 GHz, and video transmission was performed at an RF frequency of 28 GHz using a beamforming antenna. Similarly, the receiver used a beamforming antenna to receive the 28 GHz RF signal, and like the transmitter, utilized the transceiver module to down-convert the received signals to 802 MHz. A DVB-T2 receiver was utilized to receive the signal from the transceiver module and displayed the received video in the device. The experimental setup for real-time wireless video transmission at a distance of 15 m is shown in Figure 20a. To assess the effectiveness of the phased-array system, a video transmission test was conducted, maintaining a stationary transmitting location while moving the receiver in different directions. Remarkably, the video was successfully transmitted without interruption within a range of ±50°. The phase and gain of each of the eight channels were fine-tuned via SPI. The transmitted OFDM measurement spectrum is illustrated in Figure 21, while the received OFDM measurement spectrum is presented in Figure 22. The system displayed image rejection of nearly 30 dB, confirming the effectiveness of the phased-array beamforming system.

## 4. Conclusions

In this paper, we present a 5G NR FR2 beamforming system with an integrated transceiver module. This system facilitates simultaneous data transmission and reception using the same module. The incorporation of an eight-channel custom-made BFIC in the phased-array antenna system significantly contributes to enhanced performance and efficiency. The beamforming antenna exhibits the horizontal scanning ability from −50° to +50° with 10° intervals. Furthermore, the integrated transceiver module offers flexibility in adjusting the frequencies of both the local oscillator (LO) and intermediate frequency (IF) with minimal complexity. The transceiver module achieves a transmission and reception conversion gain of approximately 20 dB. In this work, we demonstrate a circuit to the system. The integration of our own eight-channel BFIC, array antenna, and our own measurement setup enables the effective demonstration of a 5G mm-wave system. The feasibility of real-time video transmission in various directions using the beamforming system is successfully showcased, highlighting its applicability in diverse equipment, particularly in radar, mobile, and satellite communications.

## Figures and Tables

**Table 1:** Summary of 28 GHz RF beamforming system performance.

Ref.	This Work	[4]	[27]	[28]
Number of elements	8 × 7	4 × 2	4 × 2	8 × 8
Freq. (GHz)	28	25.8–28	26.5–29.5	28–32
Polarization	Single	Dual	Dual	Single
Gain (dBi)	15	14	N/A	22.5
Scan range (°)	±50	±20	±45	±50
HPBW (°)	13	N/A	N/A	10.5
SRR (dB)	15	9	7	10

## Data Availability

The data can be obtained from the authors on request.
